# Probability-based model of protein-protein interactions on biological timescales

**DOI:** 10.1186/1748-7188-1-25

**Published:** 2006-12-11

**Authors:** Alexander L Tournier, Paul W Fitzjohn, Paul A Bates

**Affiliations:** 1 Biomolecular Modelling Laboratory, Cancer Research UK London Research Institute, 44 Lincoln's Inn Fields, London WC2A 3PX, UK

## Abstract

**Background:**

Simulation methods can assist in describing and understanding complex networks of interacting proteins, providing fresh insights into the function and regulation of biological systems. Recent studies have investigated such processes by explicitly modelling the diffusion and interactions of individual molecules. In these approaches, two entities are considered to have interacted if they come within a set cutoff distance of each other.

**Results:**

In this study, a new model of bimolecular interactions is presented that uses a simple, probability-based description of the reaction process. This description is well-suited to simulations on timescales relevant to biological systems (from seconds to hours), and provides an alternative to the previous description given by Smoluchowski. In the present approach (TFB) the diffusion process is explicitly taken into account in generating the probability that two freely diffusing chemical entities will interact within a given time interval. It is compared to the Smoluchowski method, as modified by Andrews and Bray (AB).

**Conclusion:**

When implemented, the AB & TFB methods give equivalent results in a variety of situations relevant to biology. Overall, the Smoluchowski method as modified by Andrews and Bray emerges as the most simple, robust and efficient method for simulating biological diffusion-reaction processes currently available.

## Background

Molecular biology is moving to an age where the amount of data and its complexity challenge our efforts to understand it. Many recent experimental studies have concentrated on obtaining accurate protein-protein interaction maps for genomes, ranging from unicellular organisms to human. Combining experimental data with modelling makes it possible to tackle this new wealth of information and study the way function emerges from protein interaction networks (for reviews of this field see references [1-3]).

An effective approach to simulating interaction networks, and one which has been used extensively in building cellular models, is through the use of ordinary differential equations (ODEs) (see review by Tyson *etal *[4] and references therein). ODEs, however, suffer from two important limitations.

The first limitation is that they are designed to follow the bulk concentration of the different molecules. In many cases, where small quantities of molecules are involved, the dynamics of the system are known to deviate substantially from the deterministic prediction of the ODEs and are better described by stochastic laws [5]. This can be overcome by implementing stochasticity into the models, which can be achieved in three ways: a first way is to use ODEs where stochastic perturbations have been added, mimicking the way the concentration of molecules fluctuates in time [6]; a second way is to use the method developed by Gillespie which follows reactions as discreet events in time [7]; and a third way – the one taken in the present work – is to explicitly follow the state of all the different molecules in the system independently [8].

A second limitation of the ODE approach, and of subsequent stochastic improvements, is that diffusion is not explicitly taken into account, which means that the effect of concentration gradients cannot be followed [9-11]. Concentration gradients can themselves be modelled, however it then becomes problematic to include the stochastic components (Virtual Cell approach [12-14] and E-Cell [15,16]).

One way of modelling the stochastic as well as the diffusive aspect of the problem is by explicitly modelling the diffusion and interactions of the individual molecules contained in the system. Such spatial simulations have been performed by Franks *etal*. to study the synaptic cleft using their software M-Cell [10]. Also, recent simulations by Lipkow *etal*. have successfully modelled the individual molecules and their diffusion to show the presence of a protein concentration gradient in the motor response in *Escherichia coli *using their software *SmolDyn *[17,18]. Another way is to discretise space on a lattice and to use extensions of the Gillespie algorithm such as in SmartCell [11,19] and MesoRD [20,21].

Bimolecular interactions have previously been modelled by considering a simple local contact criteria, such a scheme is used in M-Cell [10]. A more formal approach to modelling these interactions follows the description of diffusion limited chemical processes published by Smoluchowski in 1916 [22]. In this approach a chemical reaction is considered to take place when two chemically reactive entities A and B come within a certain distance, *σ*_*b*_, from one another. This distance, called the reaction radius, is determined by the reaction rate and the diffusion constants of the two species, such that the reaction rate, *k, *is given by:

*k *= 4 *πD*_+ _*σ*_*b *_    (1)

where *D*_+ _= *D*_*A *_+ *D*_*B*_, and *D*_*A *_and *D*_*B *_are the diffusion constants of A and B.

**Figure 1:**
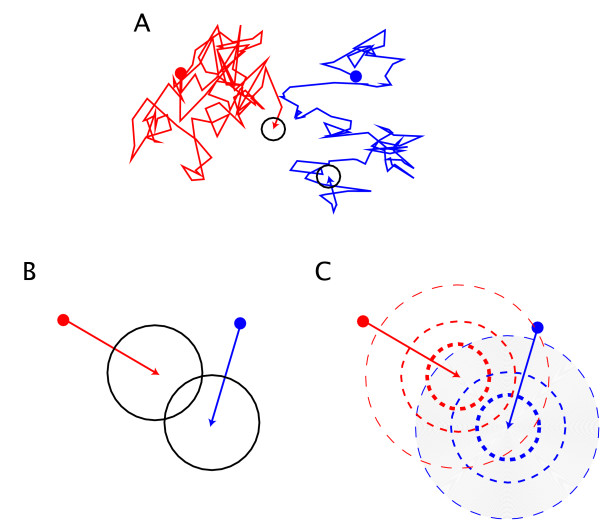
**Comparison of different approaches to modeling chemical reactions**. Comparison of different approaches to modelling chemical reactions: (A) the original Smoluchowski algorithm, (B) the corrected Andrews and Bray approach and (C) the present probability-based model. The reaction radii are shown in black in approaches (A) and (B). Probability densities are indicated by the hashed lines for approach (C).

The Smoluchowski approach requires the diffusion process to be followed using very short timesteps as the distance between the two entities must be precisely monitored over time. However, since the detailed diffusion process is of little interest in biological terms, this requirement translates into an unnecessary computational overhead, as illustrated in Figure 1. In order to circumvent this problem, Andrews and Bray recently devised a scheme which corrects *σ*_*b *_for longer timesteps, making it more useful for simulating biological systems [17].

The Smoluchowski approach seems to be the most appropriate method currently available to study many important biological systems, however a potential weakness of the Smoluchowski approach is the presence of a sharply defined reaction zone (cf. Figure 1). The aim of the present study is to investigate the potential benefit of replacing this reaction zone by a more realistic probability distribution of interaction between two chemical objects. In this scheme, the reaction is not automatic when two reacting objects come within a certain range of each other. Instead, the decision whether to allow this interaction is made based on a probability. This probability of interaction is dependent upon – among other factors – the distance between the objects. Potential benefits of the study include more accurate results and lower computational costs, thereby allowing for more complicated systems to be investigated.

The approach has been implemented into a freely available simulation package, SoftCell. In the SoftCell software cellular membranes are defined by tessellation using triangles and rates of import/export are assigned to each chemical entity. This tessellation approach makes it possible to define complicated surfaces and any number of internal organelles one might wish to include. The program is written in C++ and is linked with the scripting language, Python, allowing for control and ease of analysis of the data generated. Files defining protein objects, reactions, and membranes use an XML format.

## The model

**Figure 2:**
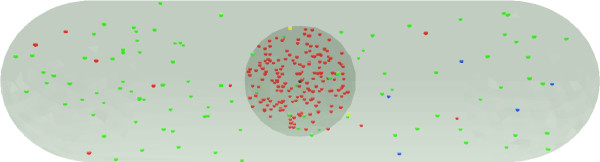
**Possible applications of the method**. An example of the kind of simulation this approach is designed for. This example illustrates a simulation of Schizosaccharomyces pombe yeast cell 10 *μ*m long. Different types of proteins are shown in different colours, each has its own diffusion, reaction and location (nuclear or cytosolic) characteristics.

In the present approach we consider proteins to be freely diffusing point-like objects. On the scale of a whole cell which is the scale we are interested in, long range forces between the objects are shielded by the solvent and can therefore safely be ignored. Diffusion is formally modelled by Brownian dynamics, taking intermolecular forces explicitly into account, and integrating over the velocities of the object [23]. In the absence of long-range forces, the Brownian dynamics treatment of diffusion reduces to a random walk process. The random walk process only considers the position of the objects and not their velocities thereby reducing considerably the computational cost; this approach was therefore used in this work. It is also assumed that any differences in reaction kinetics resulting from the different possible orientations of two reacting molecules relative to each other at the time of encounter can safely be integrated into an average reaction kinetic, so that the objects can be treated as point-like. Interactions between these point-like objects are governed by a set of reaction rules (described in detail below) that are designed to emulate the biological system of interest as closely as possible, as illustrated in Figure 2.

### Reaction rules

We are interested in the reaction probability: the probability that two entities interact during a time step given that they can interact with reaction rate *k*, their diffusion rates are *D*_1 _and *D*_2 _and they start a distance *d *apart at the beginning of the time interval Δ*t*. This probability is illustrated in Figure 1C. The reaction between two diffusing particles can be considered to occur in two steps: firstly the encounter of the two entities through diffusion, followed by the actual chemical reaction. Let us consider two freely diffusing chemical entities, A and B, starting a distance *d*_0 _from each other at time *t*_0_. At any time *t *later the rate *κ*^*AB *^(*t*|*d*_0_, *t*_0_) of the reaction between entities A and B can be expressed as:

κAB(t|d0,t0)=pCAB(t|d0,t0)⋅kRAB     (2)
 MathType@MTEF@5@5@+=feaafiart1ev1aaatCvAUfKttLearuWrP9MDH5MBPbIqV92AaeXatLxBI9gBaebbnrfifHhDYfgasaacH8akY=wiFfYdH8Gipec8Eeeu0xXdbba9frFj0=OqFfea0dXdd9vqai=hGuQ8kuc9pgc9s8qqaq=dirpe0xb9q8qiLsFr0=vr0=vr0dc8meaabaqaciaacaGaaeqabaqabeGadaaakeaaiiGacqWF6oWAdaahaaWcbeqaaiabdgeabjabdkeacbaakiabcIcaOiabdsha0jabcYha8jabdsgaKnaaBaaaleaacqaIWaamaeqaaOGaeiilaWIaemiDaq3aaSbaaSqaaiabicdaWaqabaGccqGGPaqkcqGH9aqpcqWGWbaCdaqhaaWcbaGaem4qameabaGaemyqaeKaemOqaieaaOGaeiikaGIaemiDaqNaeiiFaWNaemizaq2aaSbaaSqaaiabicdaWaqabaGccqGGSaalcqWG0baDdaWgaaWcbaGaeGimaadabeaakiabcMcaPiabgwSixlabdUgaRnaaDaaaleaacqWGsbGuaeaacqWGbbqqcqWGcbGqaaGccaWLjaGaaCzcamaabmaabaGaeGOmaidacaGLOaGaayzkaaaaaa@567F@

where pCAB
 MathType@MTEF@5@5@+=feaafiart1ev1aaatCvAUfKttLearuWrP9MDH5MBPbIqV92AaeXatLxBI9gBaebbnrfifHhDYfgasaacH8akY=wiFfYdH8Gipec8Eeeu0xXdbba9frFj0=OqFfea0dXdd9vqai=hGuQ8kuc9pgc9s8qqaq=dirpe0xb9q8qiLsFr0=vr0=vr0dc8meaabaqaciaacaGaaeqabaqabeGadaaakeaacqWGWbaCdaqhaaWcbaGaem4qameabaGaemyqaeKaemOqaieaaaaa@3169@ (*t*|*d*_0_, *t*_0_) is the probability of the two entities coming into contact at time *t *and kRAB
 MathType@MTEF@5@5@+=feaafiart1ev1aaatCvAUfKttLearuWrP9MDH5MBPbIqV92AaeXatLxBI9gBaebbnrfifHhDYfgasaacH8akY=wiFfYdH8Gipec8Eeeu0xXdbba9frFj0=OqFfea0dXdd9vqai=hGuQ8kuc9pgc9s8qqaq=dirpe0xb9q8qiLsFr0=vr0=vr0dc8meaabaqaciaacaGaaeqabaqabeGadaaakeaacqWGRbWAdaqhaaWcbaGaemOuaifabaGaemyqaeKaemOqaieaaaaa@317D@ is the rate of the reaction once in contact, averaged over all possible orientations of the two entities relative to each other. Both parts of this equation: pCAB
 MathType@MTEF@5@5@+=feaafiart1ev1aaatCvAUfKttLearuWrP9MDH5MBPbIqV92AaeXatLxBI9gBaebbnrfifHhDYfgasaacH8akY=wiFfYdH8Gipec8Eeeu0xXdbba9frFj0=OqFfea0dXdd9vqai=hGuQ8kuc9pgc9s8qqaq=dirpe0xb9q8qiLsFr0=vr0=vr0dc8meaabaqaciaacaGaaeqabaqabeGadaaakeaacqWGWbaCdaqhaaWcbaGaem4qameabaGaemyqaeKaemOqaieaaaaa@3169@ (*t*|*d*_0_, *t*_0_) and kRAB
 MathType@MTEF@5@5@+=feaafiart1ev1aaatCvAUfKttLearuWrP9MDH5MBPbIqV92AaeXatLxBI9gBaebbnrfifHhDYfgasaacH8akY=wiFfYdH8Gipec8Eeeu0xXdbba9frFj0=OqFfea0dXdd9vqai=hGuQ8kuc9pgc9s8qqaq=dirpe0xb9q8qiLsFr0=vr0=vr0dc8meaabaqaciaacaGaaeqabaqabeGadaaakeaacqWGRbWAdaqhaaWcbaGaemOuaifabaGaemyqaeKaemOqaieaaaaa@317D@ can be estimated as described below.

The reaction rate *κ*^*AB *^(*t*|*d*_0_, *t*_0_) can be integrated over a simulation timestep Δ*t *to provide the probability of at least one reaction taking place in that timestep. We are interested in the probability of at least one event taking place during the time interval Δ*t*, i.e. 1 - P(no event during Δ*t*). The process under consideration is a Poisson process with a time dependent rate of the event taking place. Given the rate *κ*(*t*) of an event taking place at a time *t*, the probability, *P*^*AB*^, of at least one reaction taking place during that time interval takes the general form [24,25]:

*P*^*AB *^(Δ*t*) = 1 - *e*^-*I*(Δ*t*) ^    (3)

where

I(Δt)=∫0Δtκ(t)dt     (4)
 MathType@MTEF@5@5@+=feaafiart1ev1aaatCvAUfKttLearuWrP9MDH5MBPbIqV92AaeXatLxBI9gBaebbnrfifHhDYfgasaacH8akY=wiFfYdH8Gipec8Eeeu0xXdbba9frFj0=OqFfea0dXdd9vqai=hGuQ8kuc9pgc9s8qqaq=dirpe0xb9q8qiLsFr0=vr0=vr0dc8meaabaqaciaacaGaaeqabaqabeGadaaakeaacqWGjbqscqGGOaakcqqHuoarcqWG0baDcqGGPaqkcqGH9aqpdaWdXaqaaGGaciab=P7aRjabcIcaOiabdsha0jabcMcaPiabbsgaKjabdsha0bWcbaGaeGimaadabaGaeuiLdqKaemiDaqhaniabgUIiYdGccaWLjaGaaCzcamaabmaabaGaeGinaqdacaGLOaGaayzkaaaaaa@44AD@

Such that the probability of a reaction taking place during timestep *δt *can be expressed as:

PAB(Δt)=1−e−∫0ΔtκAB(t)dt     (5)
 MathType@MTEF@5@5@+=feaafiart1ev1aaatCvAUfKttLearuWrP9MDH5MBPbIqV92AaeXatLxBI9gBaebbnrfifHhDYfgasaacH8akY=wiFfYdH8Gipec8Eeeu0xXdbba9frFj0=OqFfea0dXdd9vqai=hGuQ8kuc9pgc9s8qqaq=dirpe0xb9q8qiLsFr0=vr0=vr0dc8meaabaqaciaacaGaaeqabaqabeGadaaakeaacqWGqbaudaahaaWcbeqaaiabdgeabjabdkeacbaakiabcIcaOiabfs5aejabdsha0jabcMcaPiabg2da9iabigdaXiabgkHiTiabdwgaLnaaCaaaleqabaGaeyOeI0Yaa8qmaeaaiiGacqWF6oWAdaahaaadbeqaaiabdgeabjabdkeacbaaliabcIcaOiabdsha0jabcMcaPiabbsgaKjabdsha0badbaGaeGimaadabaGaeuiLdqKaemiDaqhaoiabgUIiYdaaaOGaaCzcaiaaxMaadaqadaqaaiabiwda1aGaayjkaiaawMcaaaaa@4DA9@

where *κ*^*AB *^(*t*) is given in equation (2).

### The contact probability: pCAB
 MathType@MTEF@5@5@+=feaafiart1ev1aaatCvAUfKttLearuWrP9MDH5MBPbIqV92AaeXatLxBI9gBaebbnrfifHhDYfgasaacH8akY=wiFfYdH8Gipec8Eeeu0xXdbba9frFj0=OqFfea0dXdd9vqai=hGuQ8kuc9pgc9s8qqaq=dirpe0xb9q8qiLsFr0=vr0=vr0dc8meaabaqaciaacaGaaeqabaqabeGadaaakeaacqWGWbaCdaqhaaWcbaGaem4qameabaGaemyqaeKaemOqaieaaaaa@3169@ (*t*|*d*_0_, *t*_0_)

The probability of contact, pCAB
 MathType@MTEF@5@5@+=feaafiart1ev1aaatCvAUfKttLearuWrP9MDH5MBPbIqV92AaeXatLxBI9gBaebbnrfifHhDYfgasaacH8akY=wiFfYdH8Gipec8Eeeu0xXdbba9frFj0=OqFfea0dXdd9vqai=hGuQ8kuc9pgc9s8qqaq=dirpe0xb9q8qiLsFr0=vr0=vr0dc8meaabaqaciaacaGaaeqabaqabeGadaaakeaacqWGWbaCdaqhaaWcbaGaem4qameabaGaemyqaeKaemOqaieaaaaa@3169@ (*t*|*d*_0_, *t*_0_), is determined by the diffusion process of the two entities A and B during the time interval Δ*t *= *t *- *t*_0_. The interacting bodies follow the laws of diffusion such that the probability of finding a given entity in an infinitesimal volume element *dV*, a distance *d *away from its starting position a time Δ*t *later, is given by the well known Gaussian distribution: (4πDΔt)−3/2e−d24DΔtdV
 MathType@MTEF@5@5@+=feaafiart1ev1aaatCvAUfKttLearuWrP9MDH5MBPbIqV92AaeXatLxBI9gBaebbnrfifHhDYfgasaacH8akY=wiFfYdH8Gipec8Eeeu0xXdbba9frFj0=OqFfea0dXdd9vqai=hGuQ8kuc9pgc9s8qqaq=dirpe0xb9q8qiLsFr0=vr0=vr0dc8meaabaqaciaacaGaaeqabaqabeGadaaakeaacqGGOaakcqaI0aaniiGacqWFapaCcqWGebarcqqHuoarcqWG0baDcqGGPaqkdaahaaWcbeqaaiabgkHiTiabiodaZiabc+caViabikdaYaaakiabdwgaLnaaCaaaleqabaGaeyOeI0YaaSaaaeaacqWGKbazdaahaaadbeqaaiabikdaYaaaaSqaaiabisda0iabdseaejabfs5aejabdsha0baaaaGccqWGKbazcqWGwbGvaaa@4557@, where *D *is the diffusion constant of the entity [26].

**Figure 3:**
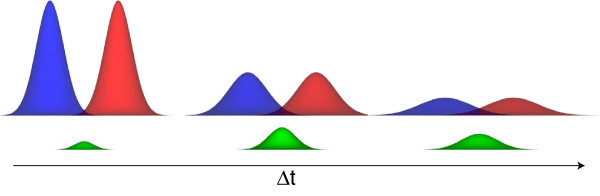
**Probability density functions**. The diffusion of the two entities A and B (red and blue) gives rise to a certain probability of them coming into contact (green). The two entities diffuse freely starting a distance *d *apart. As time goes by the probability of them coming into contact increases and reaches a maximum. Subsequently the two entities diffuse further and the probability of them coming into contact decreases with time (note: this is a 1D projection of the probability density profile, in 3D the integral over the probability density is correctly normalised to 1).

The present approach is illustrated in Figure 3. The two entities diffuse freely starting a distance *d*_0 _apart. The probability of them coming into contact increases with time and reaches a maximum. Subsequently the two entities diffuse further and the probability of them coming into contact decreases with time. A mathematically equivalent description is given if A is considered to be diffusing with diffusion constant *D*_+ _= *D*_*A *_+ *D*_*B *_while B remains stationary. It follows that the probability of contact, pCAB
 MathType@MTEF@5@5@+=feaafiart1ev1aaatCvAUfKttLearuWrP9MDH5MBPbIqV92AaeXatLxBI9gBaebbnrfifHhDYfgasaacH8akY=wiFfYdH8Gipec8Eeeu0xXdbba9frFj0=OqFfea0dXdd9vqai=hGuQ8kuc9pgc9s8qqaq=dirpe0xb9q8qiLsFr0=vr0=vr0dc8meaabaqaciaacaGaaeqabaqabeGadaaakeaacqWGWbaCdaqhaaWcbaGaem4qameabaGaemyqaeKaemOqaieaaaaa@3169@ (*t*|*r*_0_, *t*_0_), is given by:

pCAB(t|d0,t0)=(4πD+Δt)−3/2e−d024D+ΔtδVC     (6)
 MathType@MTEF@5@5@+=feaafiart1ev1aaatCvAUfKttLearuWrP9MDH5MBPbIqV92AaeXatLxBI9gBaebbnrfifHhDYfgasaacH8akY=wiFfYdH8Gipec8Eeeu0xXdbba9frFj0=OqFfea0dXdd9vqai=hGuQ8kuc9pgc9s8qqaq=dirpe0xb9q8qiLsFr0=vr0=vr0dc8meaabaqaciaacaGaaeqabaqabeGadaaakeaacqWGWbaCdaqhaaWcbaGaem4qameabaGaemyqaeKaemOqaieaaOGaeiikaGIaemiDaqNaeiiFaWNaemizaq2aaSbaaSqaaiabicdaWaqabaGccqGGSaalcqWG0baDdaWgaaWcbaGaeGimaadabeaakiabcMcaPiabg2da9iabcIcaOiabisda0GGaciab=b8aWjabdseaenaaBaaaleaacqGHRaWkaeqaaOGaeuiLdqKaemiDaqNaeiykaKYaaWbaaSqabeaacqGHsislcqaIZaWmcqGGVaWlcqaIYaGmaaGccqWGLbqzdaahaaWcbeqaaiabgkHiTmaalaaabaGaemizaq2aa0baaWqaaiabicdaWaqaaiabikdaYaaaaSqaaiabisda0iabdseaenaaBaaameaacqGHRaWkaeqaaSGaeuiLdqKaemiDaqhaaaaakiab=r7aKjabdAfawnaaBaaaleaacqWGdbWqaeqaaOGaaCzcaiaaxMaadaqadaqaaiabiAda2aGaayjkaiaawMcaaaaa@5E2C@

where *D*_+ _= *D*_*A *_+ *D*_*B*_, Δ*t *= *t *- *t*_0 _and *δV*_*C *_is a small contact volume defined such that if two entities are found to be within this volume, they are considered to be in contact. This small contact volume, *δV*_*C*_, will be considered further below.

### The reaction rate: kRAB
 MathType@MTEF@5@5@+=feaafiart1ev1aaatCvAUfKttLearuWrP9MDH5MBPbIqV92AaeXatLxBI9gBaebbnrfifHhDYfgasaacH8akY=wiFfYdH8Gipec8Eeeu0xXdbba9frFj0=OqFfea0dXdd9vqai=hGuQ8kuc9pgc9s8qqaq=dirpe0xb9q8qiLsFr0=vr0=vr0dc8meaabaqaciaacaGaaeqabaqabeGadaaakeaacqWGRbWAdaqhaaWcbaGaemOuaifabaGaemyqaeKaemOqaieaaaaa@317D@

In order to get a good first approximation for pCAB
 MathType@MTEF@5@5@+=feaafiart1ev1aaatCvAUfKttLearuWrP9MDH5MBPbIqV92AaeXatLxBI9gBaebbnrfifHhDYfgasaacH8akY=wiFfYdH8Gipec8Eeeu0xXdbba9frFj0=OqFfea0dXdd9vqai=hGuQ8kuc9pgc9s8qqaq=dirpe0xb9q8qiLsFr0=vr0=vr0dc8meaabaqaciaacaGaaeqabaqabeGadaaakeaacqWGWbaCdaqhaaWcbaGaem4qameabaGaemyqaeKaemOqaieaaaaa@3169@ (*t*|*d*_0_, *t*_0_), we initially consider the well-mixed limit. In this limit the distribution of the two entities A and B is uniform over space. This approximation is thus only valid in the long timestep limit. For short timesteps, the approximations break down and a correction to the reaction rate, kRAB
 MathType@MTEF@5@5@+=feaafiart1ev1aaatCvAUfKttLearuWrP9MDH5MBPbIqV92AaeXatLxBI9gBaebbnrfifHhDYfgasaacH8akY=wiFfYdH8Gipec8Eeeu0xXdbba9frFj0=OqFfea0dXdd9vqai=hGuQ8kuc9pgc9s8qqaq=dirpe0xb9q8qiLsFr0=vr0=vr0dc8meaabaqaciaacaGaaeqabaqabeGadaaakeaacqWGRbWAdaqhaaWcbaGaemOuaifabaGaemyqaeKaemOqaieaaaaa@317D@, has to be introduced. An exact analytical solution has recently been presented that is also correct for short time steps [27]. However, the mathematics involved are complex and difficult to implement; the approach used here, although approximate, is simpler and is not expected to alter the findings significantly.

#### The well-mixed limit

Let us consider the simple reaction: A+B→kY
 MathType@MTEF@5@5@+=feaafiart1ev1aaatCvAUfKttLearuWrP9MDH5MBPbIqV92AaeXatLxBI9gBaebbnrfifHhDYfgasaacH8akY=wiFfYdH8Gipec8Eeeu0xXdbba9frFj0=OqFfea0dXdd9vqai=hGuQ8kuc9pgc9s8qqaq=dirpe0xb9q8qiLsFr0=vr0=vr0dc8meaabaqaciaacaGaaeqabaqabeGadaaakeaacqqGbbqqcqGHRaWkcqqGcbGqdaGdKaWcbaGaem4AaSgabeGccaGLsgcacqqGzbqwaaa@33D2@ occurring in a finite volume *V*. To first order, the rate of change in the number of molecules Y is:

dnYdt=kNAnAnBV     (7)
 MathType@MTEF@5@5@+=feaafiart1ev1aaatCvAUfKttLearuWrP9MDH5MBPbIqV92AaeXatLxBI9gBaebbnrfifHhDYfgasaacH8akY=wiFfYdH8Gipec8Eeeu0xXdbba9frFj0=OqFfea0dXdd9vqai=hGuQ8kuc9pgc9s8qqaq=dirpe0xb9q8qiLsFr0=vr0=vr0dc8meaabaqaciaacaGaaeqabaqabeGadaaakeaadaWcaaqaaiabbsgaKjabd6gaUnaaBaaaleaacqqGzbqwaeqaaaGcbaGaeeizaqMaemiDaqhaaiabg2da9maalaaabaGaem4AaSgabaGaemOta40aaSbaaSqaaiabdgeabbqabaaaaOWaaSaaaeaacqWGUbGBdaWgaaWcbaGaeeyqaeeabeaakiabd6gaUnaaBaaaleaacqqGcbGqaeqaaaGcbaGaemOvayfaaiaaxMaacaWLjaWaaeWaaeaacqaI3aWnaiaawIcacaGLPaaaaaa@42D2@

where *n*_A_, *n*_B _and *n*_Y _are the number of molecules of A, B and Y (respectively), *N*_*A *_is Avogadro's constant and k is rate of the reaction. The rate of change in *n*_Y _can also be expressed as:

dnYdt=p^CAB⋅kRAB     (8)
 MathType@MTEF@5@5@+=feaafiart1ev1aaatCvAUfKttLearuWrP9MDH5MBPbIqV92AaeXatLxBI9gBaebbnrfifHhDYfgasaacH8akY=wiFfYdH8Gipec8Eeeu0xXdbba9frFj0=OqFfea0dXdd9vqai=hGuQ8kuc9pgc9s8qqaq=dirpe0xb9q8qiLsFr0=vr0=vr0dc8meaabaqaciaacaGaaeqabaqabeGadaaakeaadaWcaaqaaiabbsgaKjabd6gaUnaaBaaaleaacqqGzbqwaeqaaaGcbaGaeeizaqMaemiDaqhaaiabg2da9iqbdchaWzaajaWaa0baaSqaaiabdoeadbqaaiabdgeabjabdkeacbaakiabgwSixlabdUgaRnaaDaaaleaacqWGsbGuaeaacqWGbbqqcqWGcbGqaaGccaWLjaGaaCzcamaabmaabaGaeGioaGdacaGLOaGaayzkaaaaaa@446C@

where p^CAB
 MathType@MTEF@5@5@+=feaafiart1ev1aaatCvAUfKttLearuWrP9MDH5MBPbIqV92AaeXatLxBI9gBaebbnrfifHhDYfgasaacH8akY=wiFfYdH8Gipec8Eeeu0xXdbba9frFj0=OqFfea0dXdd9vqai=hGuQ8kuc9pgc9s8qqaq=dirpe0xb9q8qiLsFr0=vr0=vr0dc8meaabaqaciaacaGaaeqabaqabeGadaaakeaacuWGWbaCgaqcamaaDaaaleaacqWGdbWqaeaacqWGbbqqcqWGcbGqaaaaaa@3179@ is here the ensemble average probability of any two entities A and B being in contact in volume *V *and kRAB
 MathType@MTEF@5@5@+=feaafiart1ev1aaatCvAUfKttLearuWrP9MDH5MBPbIqV92AaeXatLxBI9gBaebbnrfifHhDYfgasaacH8akY=wiFfYdH8Gipec8Eeeu0xXdbba9frFj0=OqFfea0dXdd9vqai=hGuQ8kuc9pgc9s8qqaq=dirpe0xb9q8qiLsFr0=vr0=vr0dc8meaabaqaciaacaGaaeqabaqabeGadaaakeaacqWGRbWAdaqhaaWcbaGaemOuaifabaGaemyqaeKaemOqaieaaaaa@317D@ the rate of reaction if they are in contact.

The objects are considered to be uniformly distributed over the volume V such that the probability of A and B occupying the same contact volume *δV*_*C*_, p^CAB
 MathType@MTEF@5@5@+=feaafiart1ev1aaatCvAUfKttLearuWrP9MDH5MBPbIqV92AaeXatLxBI9gBaebbnrfifHhDYfgasaacH8akY=wiFfYdH8Gipec8Eeeu0xXdbba9frFj0=OqFfea0dXdd9vqai=hGuQ8kuc9pgc9s8qqaq=dirpe0xb9q8qiLsFr0=vr0=vr0dc8meaabaqaciaacaGaaeqabaqabeGadaaakeaacuWGWbaCgaqcamaaDaaaleaacqWGdbWqaeaacqWGbbqqcqWGcbGqaaaaaa@3179@, can be expressed as:

p^CAB=nAnBVδVC     (9)
 MathType@MTEF@5@5@+=feaafiart1ev1aaatCvAUfKttLearuWrP9MDH5MBPbIqV92AaeXatLxBI9gBaebbnrfifHhDYfgasaacH8akY=wiFfYdH8Gipec8Eeeu0xXdbba9frFj0=OqFfea0dXdd9vqai=hGuQ8kuc9pgc9s8qqaq=dirpe0xb9q8qiLsFr0=vr0=vr0dc8meaabaqaciaacaGaaeqabaqabeGadaaakeaacuWGWbaCgaqcamaaDaaaleaacqWGdbWqaeaacqWGbbqqcqWGcbGqaaGccqGH9aqpdaWcaaqaaiabd6gaUnaaBaaaleaacqqGbbqqaeqaaOGaemOBa42aaSbaaSqaaiabbkeacbqabaaakeaacqWGwbGvaaacciGae8hTdqMaemOvay1aaSbaaSqaaiabdoeadbqabaGccaWLjaGaaCzcamaabmaabaGaeGyoaKdacaGLOaGaayzkaaaaaa@410B@

where *δV*_*C *_is the same infinitesimal volume as in equation (6). By combining equations (7), (8), and (9), we can extract the rate of the reaction if A and B are in contact:

kR,mixedAB=kNAδVC     (10)
 MathType@MTEF@5@5@+=feaafiart1ev1aaatCvAUfKttLearuWrP9MDH5MBPbIqV92AaeXatLxBI9gBaebbnrfifHhDYfgasaacH8akY=wiFfYdH8Gipec8Eeeu0xXdbba9frFj0=OqFfea0dXdd9vqai=hGuQ8kuc9pgc9s8qqaq=dirpe0xb9q8qiLsFr0=vr0=vr0dc8meaabaqaciaacaGaaeqabaqabeGadaaakeaacqWGRbWAdaqhaaWcbaGaemOuaiLaeiilaWIaeeyBa0MaeeyAaKMaeeiEaGNaeeyzauMaeeizaqgabaGaemyqaeKaemOqaieaaOGaeyypa0ZaaSaaaeaacqWGRbWAaeaacqWGobGtdaWgaaWcbaGaemyqaeeabeaaiiGakiab=r7aKjabdAfawnaaBaaaleaacqWGdbWqaeqaaaaakiaaxMaacaWLjaWaaeWaaeaacqaIXaqmcqaIWaamaiaawIcacaGLPaaaaaa@46E4@

Combining equation (10) and pCAB
 MathType@MTEF@5@5@+=feaafiart1ev1aaatCvAUfKttLearuWrP9MDH5MBPbIqV92AaeXatLxBI9gBaebbnrfifHhDYfgasaacH8akY=wiFfYdH8Gipec8Eeeu0xXdbba9frFj0=OqFfea0dXdd9vqai=hGuQ8kuc9pgc9s8qqaq=dirpe0xb9q8qiLsFr0=vr0=vr0dc8meaabaqaciaacaGaaeqabaqabeGadaaakeaacqWGWbaCdaqhaaWcbaGaem4qameabaGaemyqaeKaemOqaieaaaaa@3169@ from equation (6) into equation (2), the rate of the reaction between entities A and B, starting a distance *d*_0 _apart at a time *t *later, is given by:

κmixedAB(t|d0,t0)=(4πD+Δt)−3/2e−d024D+Δt⋅kNA     (11)
 MathType@MTEF@5@5@+=feaafiart1ev1aaatCvAUfKttLearuWrP9MDH5MBPbIqV92AaeXatLxBI9gBaebbnrfifHhDYfgasaacH8akY=wiFfYdH8Gipec8Eeeu0xXdbba9frFj0=OqFfea0dXdd9vqai=hGuQ8kuc9pgc9s8qqaq=dirpe0xb9q8qiLsFr0=vr0=vr0dc8meaabaqaciaacaGaaeqabaqabeGadaaakeaaiiGacqWF6oWAdaqhaaWcbaGaeeyBa0MaeeyAaKMaeeiEaGNaeeyzauMaeeizaqgabaGaemyqaeKaemOqaieaaOGaeiikaGIaemiDaqNaeiiFaWNaemizaq2aaSbaaSqaaiabicdaWaqabaGccqGGSaalcqWG0baDdaWgaaWcbaGaeGimaadabeaakiabcMcaPiabg2da9iabcIcaOiabisda0iab=b8aWjabdseaenaaBaaaleaacqGHRaWkaeqaaOGaeuiLdqKaemiDaqNaeiykaKYaaWbaaSqabeaacqGHsislcqaIZaWmcqGGVaWlcqaIYaGmaaGccqWGLbqzdaahaaWcbeqaaiabgkHiTmaalaaabaGaemizaq2aa0baaWqaaiabicdaWaqaaiabikdaYaaaaSqaaiabisda0iabdseaenaaBaaameaacqGHRaWkaeqaaSGaeuiLdqKaemiDaqhaaaaakiabgwSixpaalaaabaGaem4AaSgabaGaemOta40aaSbaaSqaaiabdgeabbqabaaaaOGaaCzcaiaaxMaadaqadaqaaiabigdaXiabigdaXaGaayjkaiaawMcaaaaa@671D@

In doing this, notice that the contact volume *δV*_*C *_cancels out of the equations. This effectively removes any information about the size of the particles from subsequent considerations.

Inserting kmixedAB
 MathType@MTEF@5@5@+=feaafiart1ev1aaatCvAUfKttLearuWrP9MDH5MBPbIqV92AaeXatLxBI9gBaebbnrfifHhDYfgasaacH8akY=wiFfYdH8Gipec8Eeeu0xXdbba9frFj0=OqFfea0dXdd9vqai=hGuQ8kuc9pgc9s8qqaq=dirpe0xb9q8qiLsFr0=vr0=vr0dc8meaabaqaciaacaGaaeqabaqabeGadaaakeaacqWGRbWAdaqhaaWcbaGaeeyBa0MaeeyAaKMaeeiEaGNaeeyzauMaeeizaqgabaGaemyqaeKaemOqaieaaaaa@3721@ from equation (11) into equation (4), *I*(Δ*t*) can be solved analytically using the standard integral:

∫0Δtt−32e−1tdt=π Erfc(1Δt)     (12)
 MathType@MTEF@5@5@+=feaafiart1ev1aaatCvAUfKttLearuWrP9MDH5MBPbIqV92AaeXatLxBI9gBaebbnrfifHhDYfgasaacH8akY=wiFfYdH8Gipec8Eeeu0xXdbba9frFj0=OqFfea0dXdd9vqai=hGuQ8kuc9pgc9s8qqaq=dirpe0xb9q8qiLsFr0=vr0=vr0dc8meaabaqaciaacaGaaeqabaqabeGadaaakeaadaWdXaqaaiabdsha0naaCaaaleqabaGaeyOeI0YaaSaaaeaacqaIZaWmaeaacqaIYaGmaaaaaaqaaiabicdaWaqaaiabfs5aejabdsha0bqdcqGHRiI8aOGaemyzau2aaWbaaSqabeaacqGHsisldaWcaaqaaiabigdaXaqaaiabdsha0baaaaGccqqGKbazcqWG0baDcqGH9aqpdaGcaaqaaGGaciab=b8aWbWcbeaakiabbccaGiabbweafjabbkhaYjabbAgaMjabbogaJnaabmaabaWaaSaaaeaacqaIXaqmaeaadaGcaaqaaiabfs5aejabdsha0bWcbeaaaaaakiaawIcacaGLPaaacaWLjaGaaCzcamaabmaabaGaeGymaeJaeGOmaidacaGLOaGaayzkaaaaaa@51C8@

where Erfc is the complimentary error function defined by:

Erfc(x)=2π∫x∞e−z2dz     (13)
 MathType@MTEF@5@5@+=feaafiart1ev1aaatCvAUfKttLearuWrP9MDH5MBPbIqV92AaeXatLxBI9gBaebbnrfifHhDYfgasaacH8akY=wiFfYdH8Gipec8Eeeu0xXdbba9frFj0=OqFfea0dXdd9vqai=hGuQ8kuc9pgc9s8qqaq=dirpe0xb9q8qiLsFr0=vr0=vr0dc8meaabaqaciaacaGaaeqabaqabeGadaaakeaacqqGfbqrcqqGYbGCcqqGMbGzcqqGJbWycqGGOaakcqWG4baEcqGGPaqkcqGH9aqpdaWcaaqaaiabikdaYaqaaGGaciab=b8aWbaadaWdXaqaaiabdwgaLnaaCaaaleqabaGaeyOeI0IaemOEaO3aaWbaaWqabeaacqaIYaGmaaaaaaWcbaGaemiEaGhabaGaeyOhIukaniabgUIiYdGccqqGKbazcqWG6bGEcaWLjaGaaCzcamaabmaabaGaeGymaeJaeG4mamdacaGLOaGaayzkaaaaaa@4A63@

such that *I*(Δ*t*) has the analytical form:

I(Δt)=kNA⋅14dπD+Erfc(d4ΔtD+)     (14)
 MathType@MTEF@5@5@+=feaafiart1ev1aaatCvAUfKttLearuWrP9MDH5MBPbIqV92AaeXatLxBI9gBaebbnrfifHhDYfgasaacH8akY=wiFfYdH8Gipec8Eeeu0xXdbba9frFj0=OqFfea0dXdd9vqai=hGuQ8kuc9pgc9s8qqaq=dirpe0xb9q8qiLsFr0=vr0=vr0dc8meaabaqaciaacaGaaeqabaqabeGadaaakeaacqWGjbqscqGGOaakcqqHuoarcqWG0baDcqGGPaqkcqGH9aqpdaWcaaqaaiabdUgaRbqaaiabd6eaonaaBaaaleaacqWGbbqqaeqaaaaakiabgwSixpaalaaabaGaeGymaedabaGaeGinaqJaemizaqgcciGae8hWdaNaemiraq0aaSbaaSqaaiabgUcaRaqabaaaaOGaeeyrauKaeeOCaiNaeeOzayMaee4yam2aaeWaaeaadaWcaaqaaiabdsgaKbqaamaakaaabaGaeGinaqJaeuiLdqKaemiDaqNaemiraq0aaSbaaSqaaiabgUcaRaqabaaabeaaaaaakiaawIcacaGLPaaacaWLjaGaaCzcamaabmaabaGaeGymaeJaeGinaqdacaGLOaGaayzkaaaaaa@5368@

Finally we can express the probability PmixedAB
 MathType@MTEF@5@5@+=feaafiart1ev1aaatCvAUfKttLearuWrP9MDH5MBPbIqV92AaeXatLxBI9gBaebbnrfifHhDYfgasaacH8akY=wiFfYdH8Gipec8Eeeu0xXdbba9frFj0=OqFfea0dXdd9vqai=hGuQ8kuc9pgc9s8qqaq=dirpe0xb9q8qiLsFr0=vr0=vr0dc8meaabaqaciaacaGaaeqabaqabeGadaaakeaacqWGqbaudaqhaaWcbaGaeeyBa0MaeeyAaKMaeeiEaGNaeeyzauMaeeizaqgabaGaemyqaeKaemOqaieaaaaa@36EB@ of a reaction taking place between entities A and B, starting a distance *d*_0 _apart, during the time interval Δ*t *as:

PmixedAB(t|d0,t0)=1−e−kNA⋅14d0πD+Erfc(d04ΔtD+)     (15)
 MathType@MTEF@5@5@+=feaafiart1ev1aaatCvAUfKttLearuWrP9MDH5MBPbIqV92AaeXatLxBI9gBaebbnrfifHhDYfgasaacH8akY=wiFfYdH8Gipec8Eeeu0xXdbba9frFj0=OqFfea0dXdd9vqai=hGuQ8kuc9pgc9s8qqaq=dirpe0xb9q8qiLsFr0=vr0=vr0dc8meaabaqaciaacaGaaeqabaqabeGadaaakeaacqWGqbaudaqhaaWcbaGaeeyBa0MaeeyAaKMaeeiEaGNaeeyzauMaeeizaqgabaGaemyqaeKaemOqaieaaOGaeiikaGIaemiDaqNaeiiFaWNaemizaq2aaSbaaSqaaiabicdaWaqabaGccqGGSaalcqWG0baDdaWgaaWcbaGaeGimaadabeaakiabcMcaPiabg2da9iabigdaXiabgkHiTiabdwgaLnaaCaaaleqabaGaeyOeI0YaaSaaaeaacqWGRbWAaeaacqWGobGtdaWgaaadbaGaemyqaeeabeaaaaWccqGHflY1daWcaaqaaiabigdaXaqaaiabisda0iabdsgaKnaaBaaameaacqaIWaamaeqaaGGacSGae8hWdaNaemiraq0aaSbaaWqaaiabgUcaRaqabaaaaSGaeeyrauKaeeOCaiNaeeOzayMaee4yam2aaeWaaeaadaWcaaqaaiabdsgaKnaaBaaameaacqaIWaamaeqaaaWcbaWaaOaaaeaacqaI0aancqqHuoarcqWG0baDcqWGebardaWgaaadbaGaey4kaScabeaaaeqaaaaaaSGaayjkaiaawMcaaaaakiaaxMaacaWLjaWaaeWaaeaacqaIXaqmcqaI1aqnaiaawIcacaGLPaaaaaa@6942@

The probability of two chemical entities interacting in a timestep Δ*t *is thus expressed in equation (15) in terms of the reaction rate *k*, the sum of the diffusion constants of the two entities *D*_+ _and the time interval Δ*t*. This approach provides a good description of the interactions in terms of the underlying diffusion process and reactivity of the two entities.

#### Short timestep correction

The equations above hold for situations where the system can be considered to be well-mixed. However, this assumption breaks down for small timesteps: as chemical entities react over time, there tend to be fewer potentially interacting partners close to each other so that the distribution of the two entities is no longer uniform. In the long timescale limit this is not a problem as the system is well-mixed by each diffusion step and the approximations hold. At each timestep, the reaction process creates 'dips' in the probability distribution of the entities, the spatial extent of these 'dips' is comparable to the spatial extent of the probability of reaction. In order to remain well-mixed the distance covered by one step of diffusion must be greater than the spatial extent of the 'dips' created by the reaction process. For diffusion constants typical of biomolecules, the spatial width at half-maximum of PmixedAB
 MathType@MTEF@5@5@+=feaafiart1ev1aaatCvAUfKttLearuWrP9MDH5MBPbIqV92AaeXatLxBI9gBaebbnrfifHhDYfgasaacH8akY=wiFfYdH8Gipec8Eeeu0xXdbba9frFj0=OqFfea0dXdd9vqai=hGuQ8kuc9pgc9s8qqaq=dirpe0xb9q8qiLsFr0=vr0=vr0dc8meaabaqaciaacaGaaeqabaqabeGadaaakeaacqWGqbaudaqhaaWcbaGaeeyBa0MaeeyAaKMaeeiEaGNaeeyzauMaeeizaqgabaGaemyqaeKaemOqaieaaaaa@36EB@ (*t*|*d*_0_, *t*_0_) goes to ~0.1 *μ*m for Δ*t *of the order of seconds. Considering this distance as being covered by diffusion, this gives us a typical timescale of Δ*t *≥ 0.01 s. The system can therefore be assumed to be well-mixed for timesteps of Δ*t *≥ 0.01 s. For shorter timesteps, this effect can be corrected for, as will be shown below.

Due to the reaction process, the average concentration around a chemical entity is less than predicted by the uniform distribution. The desired rate can be derived by correcting kR,mixedAB
 MathType@MTEF@5@5@+=feaafiart1ev1aaatCvAUfKttLearuWrP9MDH5MBPbIqV92AaeXatLxBI9gBaebbnrfifHhDYfgasaacH8akY=wiFfYdH8Gipec8Eeeu0xXdbba9frFj0=OqFfea0dXdd9vqai=hGuQ8kuc9pgc9s8qqaq=dirpe0xb9q8qiLsFr0=vr0=vr0dc8meaabaqaciaacaGaaeqabaqabeGadaaakeaacqWGRbWAdaqhaaWcbaGaemOuaiLaeiilaWIaeeyBa0MaeeyAaKMaeeiEaGNaeeyzauMaeeizaqgabaGaemyqaeKaemOqaieaaaaa@392E@ by a scaling factor as follows:

kRAB=C⋅kR,mixedAB     (16)
 MathType@MTEF@5@5@+=feaafiart1ev1aaatCvAUfKttLearuWrP9MDH5MBPbIqV92AaeXatLxBI9gBaebbnrfifHhDYfgasaacH8akY=wiFfYdH8Gipec8Eeeu0xXdbba9frFj0=OqFfea0dXdd9vqai=hGuQ8kuc9pgc9s8qqaq=dirpe0xb9q8qiLsFr0=vr0=vr0dc8meaabaqaciaacaGaaeqabaqabeGadaaakeaacqWGRbWAdaqhaaWcbaGaemOuaifabaGaemyqaeKaemOqaieaaOGaeyypa0Jaem4qamKaeyyXICTaem4AaS2aa0baaSqaaiabdkfasjabcYcaSiabb2gaTjabbMgaPjabbIha4jabbwgaLjabbsgaKbqaaiabdgeabjabdkeacbaakiaaxMaacaWLjaWaaeWaaeaacqaIXaqmcqaI2aGnaiaawIcacaGLPaaaaaa@4729@

The procedure we used for doing this is very similar to that used by Andrews and Bray [17] to correct for the same effect in the Smoluchowski approach and is outlined below. More elaborate mathematical considerations of this process can be found in the recent paper by Zon and Wolde 2005 [27].

Using the rate of reaction upon encounter from equation (16), the probability of the reaction taking place after each diffusion step is now given by:

PAB(t0+Δt|d0,t0)=1−e−C⋅kNA⋅14d0πD+Erfc(d02s)     (17)
 MathType@MTEF@5@5@+=feaafiart1ev1aaatCvAUfKttLearuWrP9MDH5MBPbIqV92AaeXatLxBI9gBaebbnrfifHhDYfgasaacH8akY=wiFfYdH8Gipec8Eeeu0xXdbba9frFj0=OqFfea0dXdd9vqai=hGuQ8kuc9pgc9s8qqaq=dirpe0xb9q8qiLsFr0=vr0=vr0dc8meaabaqaciaacaGaaeqabaqabeGadaaakeaacqWGqbaudaahaaWcbeqaaiabdgeabjabdkeacbaakiabcIcaOiabdsha0naaBaaaleaacqaIWaamaeqaaOGaey4kaSIaeuiLdqKaemiDaqNaeiiFaWNaemizaq2aaSbaaSqaaiabicdaWaqabaGccqGGSaalcqWG0baDdaWgaaWcbaGaeGimaadabeaakiabcMcaPiabg2da9iabigdaXiabgkHiTiabdwgaLnaaCaaaleqabaGaeyOeI0Iaem4qamKaeyyXIC9aaSaaaeaacqWGRbWAaeaacqWGobGtdaWgaaadbaGaemyqaeeabeaaaaWccqGHflY1daWcaaqaaiabigdaXaqaaiabisda0iabdsgaKnaaBaaameaacqaIWaamaeqaaGGacSGae8hWdaNaemiraq0aaSbaaWqaaiabgUcaRaqabaaaaSGaeeyrauKaeeOCaiNaeeOzayMaee4yam2aaeWaaeaadaWcaaqaaiabdsgaKnaaBaaameaacqaIWaamaeqaaaWcbaGaeGOmaiJaem4CamhaaaGaayjkaiaawMcaaaaakiaaxMaacaWLjaWaaeWaaeaacqaIXaqmcqaI3aWnaiaawIcacaGLPaaaaaa@6704@

where Δ*t *is the timestep of the simulation and we use the substitution s=2D+Δt
 MathType@MTEF@5@5@+=feaafiart1ev1aaatCvAUfKttLearuWrP9MDH5MBPbIqV92AaeXatLxBI9gBaebbnrfifHhDYfgasaacH8akY=wiFfYdH8Gipec8Eeeu0xXdbba9frFj0=OqFfea0dXdd9vqai=hGuQ8kuc9pgc9s8qqaq=dirpe0xb9q8qiLsFr0=vr0=vr0dc8meaabaqaciaacaGaaeqabaqabeGadaaakeaacqWGZbWCcqGH9aqpdaGcaaqaaiabikdaYiabdseaenaaBaaaleaacqGHRaWkaeqaaOGaeuiLdqKaemiDaqhaleqaaaaa@352E@.

For the purposes of the correction, we are interested in average effects, so from now on we consider the average concentrations of entities A and B and not the positions of entities A and B. Let us consider the radial concentration *ρ*_*B*_(*r*, *t*) of entity B around entity A, with A considered to be static at *r *= 0, while entity B has diffusion constant *D*_+ _= *D*_*A *_+ *D*_*B*_.

The radial concentration *ρ*_*B*_(*r*, *t*) of entity B around entity A, at time *t *is propagated for a simulation timestep, Δ*t*, to give *ρ*_*B*_(*r*, *t *+ Δ*t*) using a Green's function [28]:

ρB(r,t+Δt)=∫0∞ρB(r′,t)Gs(r,r′)4πr′2dr′     (18)
 MathType@MTEF@5@5@+=feaafiart1ev1aaatCvAUfKttLearuWrP9MDH5MBPbIqV92AaeXatLxBI9gBaebbnrfifHhDYfgasaacH8akY=wiFfYdH8Gipec8Eeeu0xXdbba9frFj0=OqFfea0dXdd9vqai=hGuQ8kuc9pgc9s8qqaq=dirpe0xb9q8qiLsFr0=vr0=vr0dc8meaabaqaciaacaGaaeqabaqabeGadaaakeaaiiGacqWFbpGCdaWgaaWcbaGaemOqaieabeaakiabcIcaOiabdkhaYjabcYcaSiabdsha0jabgUcaRiabfs5aejabdsha0jabcMcaPiabg2da9maapedabaGae8xWdi3aaSbaaSqaaiabdkeacbqabaaabaGaeGimaadabaGaeyOhIukaniabgUIiYdGccqGGOaakcuWGYbGCgaqbaiabcYcaSiabdsha0jabcMcaPiabdEeahnaaBaaaleaacqWGZbWCaeqaaOGaeiikaGIaemOCaiNaeiilaWIafmOCaiNbauaacqGGPaqkcqaI0aancqWFapaCcuWGYbGCgaqbamaaCaaaleqabaGaeGOmaidaaOGaeeizaqMafmOCaiNbauaacaWLjaGaaCzcamaabmaabaGaeGymaeJaeGioaGdacaGLOaGaayzkaaaaaa@5BE3@

where the Green's function *G*_*s*_(*r*, *r'*) is given by:

Gs(r,r′)=14πrr′s2π(e−(r−r′)22s2−e−(r+r′)22s2)     (19)
 MathType@MTEF@5@5@+=feaafiart1ev1aaatCvAUfKttLearuWrP9MDH5MBPbIqV92AaeXatLxBI9gBaebbnrfifHhDYfgasaacH8akY=wiFfYdH8Gipec8Eeeu0xXdbba9frFj0=OqFfea0dXdd9vqai=hGuQ8kuc9pgc9s8qqaq=dirpe0xb9q8qiLsFr0=vr0=vr0dc8meaabaqaciaacaGaaeqabaqabeGadaaakeaacqWGhbWrdaWgaaWcbaGaem4CamhabeaakiabcIcaOiabdkhaYjabcYcaSiqbdkhaYzaafaGaeiykaKIaeyypa0ZaaSaaaeaacqaIXaqmaeaacqaI0aaniiGacqWFapaCcqWGYbGCcuWGYbGCgaqbaiabdohaZnaakaaabaGaeGOmaiJae8hWdahaleqaaaaakiabcIcaOiabdwgaLnaaCaaaleqabaGaeyOeI0YaaSaaaeaacqGGOaakcqWGYbGCcqGHsislcuWGYbGCgaqbaiabcMcaPmaaCaaameqabaGaeGOmaidaaaWcbaGaeGOmaiJaem4Cam3aaWbaaWqabeaacqaIYaGmaaaaaaaakiabgkHiTiabdwgaLnaaCaaaleqabaGaeyOeI0YaaSaaaeaacqGGOaakcqWGYbGCcqGHRaWkcuWGYbGCgaqbaiabcMcaPmaaCaaameqabaGaeGOmaidaaaWcbaGaeGOmaiJaem4Cam3aaWbaaWqabeaacqaIYaGmaaaaaaaakiabcMcaPiaaxMaacaWLjaWaaeWaaeaacqaIXaqmcqaI5aqoaiaawIcacaGLPaaaaaa@6185@

The entity A is then allowed to interact with entity B such that the new concentration of B is given by:

ρ′B(r,t+Δt)=(1−PAB(r))⋅ρB(r,t+Δt)     (20)
 MathType@MTEF@5@5@+=feaafiart1ev1aaatCvAUfKttLearuWrP9MDH5MBPbIqV92AaeXatLxBI9gBaebbnrfifHhDYfgasaacH8akY=wiFfYdH8Gipec8Eeeu0xXdbba9frFj0=OqFfea0dXdd9vqai=hGuQ8kuc9pgc9s8qqaq=dirpe0xb9q8qiLsFr0=vr0=vr0dc8meaabaqaciaacaGaaeqabaqabeGadaaakeaaiiGacuWFbpGCgaqbamaaBaaaleaacqWGcbGqaeqaaOGaeiikaGIaemOCaiNaeiilaWIaemiDaqNaey4kaSIaeuiLdqKaemiDaqNaeiykaKIaeyypa0JaeiikaGIaeGymaeJaeyOeI0Iaemiuaa1aaWbaaSqabeaacqWGbbqqcqWGcbGqaaGccqGGOaakcqWGYbGCcqGGPaqkcqGGPaqkcqGHflY1cqWFbpGCdaWgaaWcbaGaemOqaieabeaakiabcIcaOiabdkhaYjabcYcaSiabdsha0jabgUcaRiabfs5aejabdsha0jabcMcaPiaaxMaacaWLjaWaaeWaaeaacqaIYaGmcqaIWaamaiaawIcacaGLPaaaaaa@5735@

where *P*^*AB *^(*r*) is the probability of A and B interacting in the following timestep from equation (17).

The reaction step acts as a sink for the concentration of B, while the concentration of B is assumed to be constant at *r *= ∞. The long-distance equilibrium solution for *ρ*_*B*_(*r*) is known to be of the form [26,29]:

ρB(r)=1+ar     (21)
 MathType@MTEF@5@5@+=feaafiart1ev1aaatCvAUfKttLearuWrP9MDH5MBPbIqV92AaeXatLxBI9gBaebbnrfifHhDYfgasaacH8akY=wiFfYdH8Gipec8Eeeu0xXdbba9frFj0=OqFfea0dXdd9vqai=hGuQ8kuc9pgc9s8qqaq=dirpe0xb9q8qiLsFr0=vr0=vr0dc8meaabaqaciaacaGaaeqabaqabeGadaaakeaaiiGacqWFbpGCdaWgaaWcbaGaemOqaieabeaakiabcIcaOiabdkhaYjabcMcaPiabg2da9iabigdaXiabgUcaRmaalaaabaGaemyyaegabaGaemOCaihaaiaaxMaacaWLjaWaaeWaaeaacqaIYaGmcqaIXaqmaiaawIcacaGLPaaaaaa@3D24@

This allows us to solve numerically for *ρ*_*B*_(*r*, *t *+ Δ*t*) around *r *= 0 while using an analytical extension for long distances (long distance was defined as *r *> *r*_*P*_, *r*_*P *_such that *P*^*AB *^(*r*) = 10^-6^). Equation (18) is then split into a numerical and an analytical part:

ρB(r,t+Δt)=∫0rPρB(r′,t)Gs(r,r′)4πr′2dr′+∫rP∞ρBana(r′,t)Gs(r,r′)4πr′2dr′     (22)
 MathType@MTEF@5@5@+=feaafiart1ev1aaatCvAUfKttLearuWrP9MDH5MBPbIqV92AaeXatLxBI9gBaebbnrfifHhDYfgasaacH8akY=wiFfYdH8Gipec8Eeeu0xXdbba9frFj0=OqFfea0dXdd9vqai=hGuQ8kuc9pgc9s8qqaq=dirpe0xb9q8qiLsFr0=vr0=vr0dc8meaabaqaciaacaGaaeqabaqabeGadaaakeaaiiGacqWFbpGCdaWgaaWcbaGaemOqaieabeaakiabcIcaOiabdkhaYjabcYcaSiabdsha0jabgUcaRiabfs5aejabdsha0jabcMcaPiabg2da9maapedabaGae8xWdi3aaSbaaSqaaiabdkeacbqabaaabaGaeGimaadabaGaemOCai3aaSbaaWqaaiabdcfaqbqabaaaniabgUIiYdGccqGGOaakcuWGYbGCgaqbaiabcYcaSiabdsha0jabcMcaPiabdEeahnaaBaaaleaacqWGZbWCaeqaaOGaeiikaGIaemOCaiNaeiilaWIafmOCaiNbauaacqGGPaqkcqaI0aancqWFapaCcuWGYbGCgaqbamaaCaaaleqabaGaeGOmaidaaOGaeeizaqMafmOCaiNbauaacqGHRaWkdaWdXaqaaiab=f8aYnaaDaaaleaacqWGcbGqaeaacqqGHbqycqqGUbGBcqqGHbqyaaaabaGaemOCai3aaSbaaWqaaiabdcfaqbqabaaaleaacqGHEisPa0Gaey4kIipakiabcIcaOiqbdkhaYzaafaGaeiilaWIaemiDaqNaeiykaKIaem4raC0aaSbaaSqaaiabdohaZbqabaGccqGGOaakcqWGYbGCcqGGSaalcuWGYbGCgaqbaiabcMcaPiabisda0iab=b8aWjqbdkhaYzaafaWaaWbaaSqabeaacqaIYaGmaaGccqqGKbazcuWGYbGCgaqbaiaaxMaacaWLjaWaaeWaaeaacqaIYaGmcqaIYaGmaiaawIcacaGLPaaaaaa@8126@

ρB(r,t+Δt)=∫0rPρB(r′,t)Gs(r,r′)4πr′2dr′+ρBana(r,t+Δt)     (23)
 MathType@MTEF@5@5@+=feaafiart1ev1aaatCvAUfKttLearuWrP9MDH5MBPbIqV92AaeXatLxBI9gBaebbnrfifHhDYfgasaacH8akY=wiFfYdH8Gipec8Eeeu0xXdbba9frFj0=OqFfea0dXdd9vqai=hGuQ8kuc9pgc9s8qqaq=dirpe0xb9q8qiLsFr0=vr0=vr0dc8meaabaqaciaacaGaaeqabaqabeGadaaakeaaiiGacqWFbpGCdaWgaaWcbaGaemOqaieabeaakiabcIcaOiabdkhaYjabcYcaSiabdsha0jabgUcaRiabfs5aejabdsha0jabcMcaPiabg2da9maapedabaGae8xWdi3aaSbaaSqaaiabdkeacbqabaaabaGaeGimaadabaGaemOCai3aaSbaaWqaaiabdcfaqbqabaaaniabgUIiYdGccqGGOaakcuWGYbGCgaqbaiabcYcaSiabdsha0jabcMcaPiabdEeahnaaBaaaleaacqWGZbWCaeqaaOGaeiikaGIaemOCaiNaeiilaWIafmOCaiNbauaacqGGPaqkcqaI0aancqWFapaCcuWGYbGCgaqbamaaCaaaleqabaGaeGOmaidaaOGaeeizaqMafmOCaiNbauaacqGHRaWkcqWFbpGCdaqhaaWcbaGaemOqaieabaGaeeyyaeMaeeOBa4MaeeyyaegaaOGaeiikaGIaemOCaiNaeiilaWIaemiDaqNaey4kaSIaeuiLdqKaemiDaqNaeiykaKIaaCzcaiaaxMaadaqadaqaaiabikdaYiabiodaZaGaayjkaiaawMcaaaaa@6E2C@

By inserting (21), the analytical extension, ρBana
 MathType@MTEF@5@5@+=feaafiart1ev1aaatCvAUfKttLearuWrP9MDH5MBPbIqV92AaeXatLxBI9gBaebbnrfifHhDYfgasaacH8akY=wiFfYdH8Gipec8Eeeu0xXdbba9frFj0=OqFfea0dXdd9vqai=hGuQ8kuc9pgc9s8qqaq=dirpe0xb9q8qiLsFr0=vr0=vr0dc8meaabaqaciaacaGaaeqabaqabeGadaaakeaaiiGacqWFbpGCdaqhaaWcbaGaemOqaieabaGaeeyyaeMaeeOBa4Maeeyyaegaaaaa@33A2@ (*r*, *t *+ Δ*t*), can be derived and is given by:

ρBana(r,t+Δt)=sr2πGs(r,rP)+12(E++E−)+a2r(E−−E+)     (24)
 MathType@MTEF@5@5@+=feaafiart1ev1aaatCvAUfKttLearuWrP9MDH5MBPbIqV92AaeXatLxBI9gBaebbnrfifHhDYfgasaacH8akY=wiFfYdH8Gipec8Eeeu0xXdbba9frFj0=OqFfea0dXdd9vqai=hGuQ8kuc9pgc9s8qqaq=dirpe0xb9q8qiLsFr0=vr0=vr0dc8meaabaqaciaacaGaaeqabaqabeGadaaakeaaiiGacqWFbpGCdaqhaaWcbaGaemOqaieabaGaeeyyaeMaeeOBa4MaeeyyaegaaOGaeiikaGIaemOCaiNaeiilaWIaemiDaqNaey4kaSIaeuiLdqKaemiDaqNaeiykaKIaeyypa0ZaaSaaaeaacqWGZbWCaeaacqWGYbGCdaGcaaqaaiabikdaYiab=b8aWbWcbeaaaaGccqWGhbWrdaWgaaWcbaGaem4CamhabeaakiabcIcaOiabdkhaYjabcYcaSiabdkhaYnaaBaaaleaacqWGqbauaeqaaOGaeiykaKIaey4kaSYaaSaaaeaacqaIXaqmaeaacqaIYaGmaaGaeiikaGIaemyrau0aaSbaaSqaaiabgUcaRaqabaGccqGHRaWkcqWGfbqrdaWgaaWcbaGaeyOeI0cabeaakiabcMcaPiabgUcaRmaalaaabaGaemyyaegabaGaeGOmaiJaemOCaihaaiabcIcaOiabdweafnaaBaaaleaacqGHsislaeqaaOGaeyOeI0Iaemyrau0aaSbaaSqaaiabgUcaRaqabaGccqGGPaqkcaWLjaGaaCzcamaabmaabaGaeGOmaiJaeGinaqdacaGLOaGaayzkaaaaaa@6737@

where: E±=Erfc(rP±rs2)
 MathType@MTEF@5@5@+=feaafiart1ev1aaatCvAUfKttLearuWrP9MDH5MBPbIqV92AaeXatLxBI9gBaebbnrfifHhDYfgasaacH8akY=wiFfYdH8Gipec8Eeeu0xXdbba9frFj0=OqFfea0dXdd9vqai=hGuQ8kuc9pgc9s8qqaq=dirpe0xb9q8qiLsFr0=vr0=vr0dc8meaabaqaciaacaGaaeqabaqabeGadaaakeaacqWGfbqrdaWgaaWcbaGaeyySaelabeaakiabg2da9iabbweafjabbkhaYjabbAgaMjabbogaJnaabmaabaWaaSaaaeaacqWGYbGCdaWgaaWcbaGaemiuaafabeaakiabgglaXkabdkhaYbqaaiabdohaZnaakaaabaGaeGOmaidaleqaaaaaaOGaayjkaiaawMcaaaaa@404B@

A diffusion step is performed using numerical integration for the remainder of equation (23). The value of the constant *a *in ρBana
 MathType@MTEF@5@5@+=feaafiart1ev1aaatCvAUfKttLearuWrP9MDH5MBPbIqV92AaeXatLxBI9gBaebbnrfifHhDYfgasaacH8akY=wiFfYdH8Gipec8Eeeu0xXdbba9frFj0=OqFfea0dXdd9vqai=hGuQ8kuc9pgc9s8qqaq=dirpe0xb9q8qiLsFr0=vr0=vr0dc8meaabaqaciaacaGaaeqabaqabeGadaaakeaaiiGacqWFbpGCdaqhaaWcbaGaemOqaieabaGaeeyyaeMaeeOBa4Maeeyyaegaaaaa@33A2@ (*r*, *t*) is found by fitting the last 10% of *ρ*_*B*_(*r*, *t*) relative to *r*_*P *_after each diffusion step. After each diffusion step the two entities are allowed to interact following the probability given in equation (17). In order to achieve a steady state, entity A is left unaffected by the reaction process. The process of diffusion/reaction is repeated until the radial concentration *ρ*_*B*_(*r*, *t*) reaches a steady state: *ρ*_*B*_(*r*, ∞).

The effective rate of the reaction is given by:

keff=∫0∞PAB(r)ρB(r,∞)4πr2dr     (25)
 MathType@MTEF@5@5@+=feaafiart1ev1aaatCvAUfKttLearuWrP9MDH5MBPbIqV92AaeXatLxBI9gBaebbnrfifHhDYfgasaacH8akY=wiFfYdH8Gipec8Eeeu0xXdbba9frFj0=OqFfea0dXdd9vqai=hGuQ8kuc9pgc9s8qqaq=dirpe0xb9q8qiLsFr0=vr0=vr0dc8meaabaqaciaacaGaaeqabaqabeGadaaakeaacqWGRbWAdaWgaaWcbaGaeeyzauMaeeOzayMaeeOzaygabeaakiabg2da9maapedabaGaemiuaa1aaWbaaSqabeaacqWGbbqqcqWGcbGqaaaabaGaeGimaadabaGaeyOhIukaniabgUIiYdGccqGGOaakcqWGYbGCcqGGPaqkiiGacqWFbpGCdaWgaaWcbaGaemOqaieabeaakiabcIcaOiabdkhaYjabcYcaSiabg6HiLkabcMcaPiabisda0iab=b8aWjabdkhaYnaaCaaaleqabaGaeGOmaidaaOGaeeizaqMaemOCaiNaaCzcaiaaxMaadaqadaqaaiabikdaYiabiwda1aGaayjkaiaawMcaaaaa@5383@

The values of *k*_eff _were determined for a range of values of the correction factor, *C*, and variable *s*. Using this array of data it is then possible to find the correct value of the correction factor, *C*, for any pair of values of the desired reaction rate and simulation timestep: (*k*_eff_, Δ*t*).

**Figure 4:**
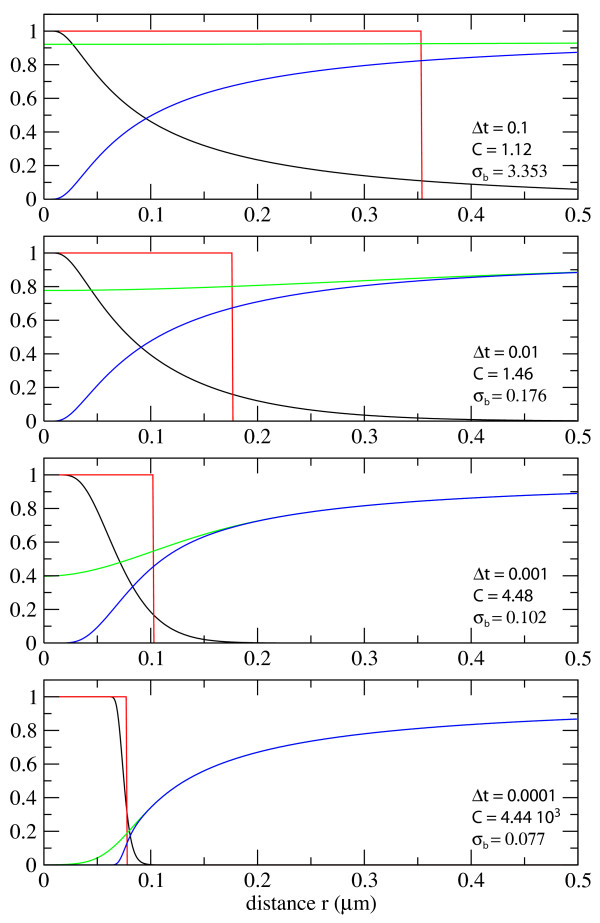
**Radial probability densities and the correction factor *C***. The radial probability distribution, *ρ*_*B, *_before interaction with A (green), and after (blue). The blue distribution evolves to the green distribution after one step of diffusion. The probability of interaction is shown in black. The probability of interaction as given by the corrected Smoluchowski approach of Andrews and Bray is also shown in red (*P*(*r*) = 1 for *r *<*σ*_*b*_). Four timesteps are shown: Δ*t *= 0.1s, 0.01s, 0.001s, 0.0001s. The correction factors and corrected Smoluchowski binding radii, *σ*_*b*_, corresponding to the different timesteps are also shown, *k *= 10^6 ^M^-1^s^-1^, and *D*_*A *_= *D*_*B *_= 1 *μ*m^2^s^-1^.

Figure 4 shows examples of the probability distribution and corresponding reaction radius, *σ*_*b*_, using the Smoluchowski approach for different timesteps. As can be seen, at large timesteps, the distribution of B before the reaction is close to uniform and the correction factor correspondingly small. On the other hand at small timesteps, the probability distribution of *B*, before reaction, is far from uniform and the correction factor, *C*, is large. For *k *= 10^6 ^M^-1^s^-1 ^and *D*_*A *_= *D*_*B *_= 1 *μ*m^2^s^-1 ^the correction factors, *C*, are 1.12, 1.46, 4.48 and 4.44 10^3 ^for timesteps of 10^-1^s, 10^-2^s, 10^-3^s and 10^-4^s, respectively. Figure 4 also illustrates the fact that as the timesteps diminish the corrected reaction probability converges with the Smoluchowski cutoff at *σ*_*b*_. For short timesteps the two approaches appear to be equivalent.

Figure 4 also shows the radial probability distribution of the reactants for the different timesteps. These distributions are continuous throughout the reaction region. In contrast, those reported by Andrews and Bray show a strong discontinuity due to the sharply defined reaction radius [17].

### The challenge of long timesteps

Relation (15) holds only for a pair of interacting molecules. In the more general context of an actual chemical reaction, the number of potential reactive partners at any one time can be much higher. The assumption is that during a time step Δ*t *the probability of an entity interacting with its closest neighbour, *P*_closest_, is much higher than the probability of it interacting with its next nearest neighbour, *P*_next nearest_: *P*_closest _≫ *P*_next nearest_.

This condition will clearly be fulfilled if the average distance, *d*_travel_, travelled by an entity during the time interval Δ*t *is less than the average distance between particles, *d*. The average travel distance is: *d*_travel _= dtravel=6DΔt
 MathType@MTEF@5@5@+=feaafiart1ev1aaatCvAUfKttLearuWrP9MDH5MBPbIqV92AaeXatLxBI9gBaebbnrfifHhDYfgasaacH8akY=wiFfYdH8Gipec8Eeeu0xXdbba9frFj0=OqFfea0dXdd9vqai=hGuQ8kuc9pgc9s8qqaq=dirpe0xb9q8qiLsFr0=vr0=vr0dc8meaabaqaciaacaGaaeqabaqabeGadaaakeaacqWGKbazdaWgaaWcbaGaeeiDaqNaeeOCaiNaeeyyaeMaeeODayNaeeyzauMaeeiBaWgabeaakiabg2da9maakaaabaGaeGOnayJaemiraqKaeuiLdqKaemiDaqhaleqaaaaa@3C7C@, where *D *is the diffusion constant. The average interparticle distance given by: d=(VN)1/3
 MathType@MTEF@5@5@+=feaafiart1ev1aaatCvAUfKttLearuWrP9MDH5MBPbIqV92AaeXatLxBI9gBaebbnrfifHhDYfgasaacH8akY=wiFfYdH8Gipec8Eeeu0xXdbba9frFj0=OqFfea0dXdd9vqai=hGuQ8kuc9pgc9s8qqaq=dirpe0xb9q8qiLsFr0=vr0=vr0dc8meaabaqaciaacaGaaeqabaqabeGadaaakeaacqWGKbazcqGH9aqpcqGGOaakdaWcaaqaaiabdAfawbqaaiabd6eaobaacqGGPaqkdaahaaWcbeqaaiabigdaXiabc+caViabiodaZaaaaaa@3616@, where *V *is the volume and *N *is the number of particle, can also be expressed in terms of the concentration as: *d *∝ *C*^-1/3^. where *C *is the concentration. For typical biological situations, *C *≃ *μ*M ml^-1 ^and *D *≃ *μ*m^2^s^-1^, such that the condition reduces to Δ*t *<~0.002 s.

When this condition is not fulfilled, a given entity can in principle interact with several other entities at any given timestep. This will affect the probability of the reaction with any given particle as reactions are considered mutually exclusive. In principle these probabilities can be calculated at each timestep during the simulation so that the correct statistics are reproduced. However, it was decided that the resulting extension to the code would introduce extra computational overhead, without significant benefit, and was not implemented. On the other hand, simply assuming that entities can interact with at most one other entity in the following timestep, when in fact they can interact with several, leads to the simulated reaction taking place at a rate greater than the expected rate.

Another problem which emerges for long timesteps concerns boundaries: for reactions, the algorithm assumes free diffusion in the space around the entities. This assumption is mostly correct when the timestep is small but at large timestep, the chance of encountering a boundary during that timestep become significant. At that point the free diffusion assumption assumed in the previous equations breaks down leading the reaction happening too fast in the simulation. Simply put, entities close to boundaries have less volume in which to diffuse and, therefore, a higher chance of encounter than entities far from any boundary. We can expect this effect to become important when the scale of the system becomes comparable to the typical distance travelled during a timestep. For biological systems on the *μ*m scale, and chemical entities diffusing with diffusion constants ~*μ*ms^-1^, this sets an absolute upper limit on the timesteps at ~0.1 s. Properly taking into account these boundary effects is beyond the scope of the present work. However, boundary effects are not expected to play an important role as the timescale for these effects is ~0.1 s, which is a much longer timescale than the limit previously set by single particle interaction at ~0.002 s.

## Validation of the model

The model was tested in a number of ways. The first test was performed by simulating an enzymatic reaction: *A *+ *E *→ *B *+ *E*. 1000 molecules of A were simulated with 10 molecules of E and the effective reaction rate was measured by fitting the change in the concentration of *A *over time. We ran 4 sets of simulations with the following parameters for the diffusion rate *d *of the chemical objects and timestep Δ*t*: 1) d = 1, Δ*t* = 0.01; 2) d = 1, Δ*t* = 0.001; 3) d = 5, Δ*t* = 0.01; 4) d = 5, Δ*t* = 0.001. For each set of d and Δ*t*, the reaction rate *k *was successively set to 10^6.0^, 10^6.2^, 10^6.4^ and 10^6.6^ M^-1^s^-1^. For comparison purposes, these 4 sets of runs were performed using both the present model and the Andrews and Bray model. The corrected binding radius used in the Andrews and Bray approach was calculated using the code provided by the authors.

As has been pointed out by Andrews and Bray [17], in such simulations the measured rate of the reaction varies with time. This is due to the fact that the simulation starts, the local concentration gradient is not yet established, and the initial reaction rate is, therefore, higher than the desired rate as the local concentration gradient is not yet established. Subsequently, the system tends towards a steady state, and the reaction rate is correctly predicted by both methods with 99% accuracy when using the correction term. The two methods were statistically indistinguishable over the four sets of runs (slope and intercept of *k*_*measured *_= *f *(*k*_*desired*_) were identical with *p *> 0.55).

The model was also tested for reactions at low numbers of reactants (*n*_*A *_= 10) where the effective rate of the reaction becomes subject to significant stochastic fluctuations. 10000 runs were performed using both the present and corrected Smoluchowski approaches; the reaction rate was determined for each run. Again four sets of runs were performed using the same diffusion constants and timesteps as described above. The reaction rate used was 10^6 ^M^-1^s^-1^. The distribution of the reaction rates at low concentrations produced by the present and corrected Smoluchowski approaches were compared and found to be indistinguishable (*p *> 0.2 on t-test).

Finally, the present and corrected Smoluchowski approaches were also compared in a situation containing a concentration gradient. The concentration gradient was produced by a point source of a molecule A (*k *= 2 *s*^-1^) which reacts with an enzyme E ([*E*] = 40 nM) with *k*_*E *_= 10^6 ^M^-1^s^-1^. The diffusion constants and timestep parameters where again varied as previously. The gradient generated were found to be identical (*p *> 0.9 on U-test). All statistical analyses were performed using the R package [30].

## Example

In a typical cell, the concentration of a given protein inside the nucleus depends on the balance between the rate at which it is being translated from mRNA in the cytosol, the rate at which it is being transported into the nucleus and the rate at which it is being degraded. In turn, the amount of mRNA present in the cytosol depends on the rate at which the gene is being transcribed in the nucleus, the rate at which it is being exported and the rate at which it is being degraded. This mechanism enables the concentration of protein in the nucleus to be tightly controlled.

As an illustration of the versatility of the present approach this simple system was simulated. Our model system, illustrated in Figure 2, consisted of a rod-shaped cell with a nucleus at its centre. Inside the nucleus, a gene is switched on at time *t *= 0, for 20 minutes. Transcription events then take place, generating mRNA molecules. These mRNA molecules diffuse out of the nucleus and encounter ribosomes which translate them into proteins. These proteins are considered to be tagged for the nucleus and therefore are allowed to pass through the membrane and accumulate in the nucleus. Both the mRNA and the protein have ubiquitination/destruction pathways that regulate their lifetime inside the cell such that the system reaches a steady state with a finite concentration of mRNA and protein. At time *t *= 20 min the gene is turned off and the concentration of mRNA and protein drops rapidly.

**Figure 5:**
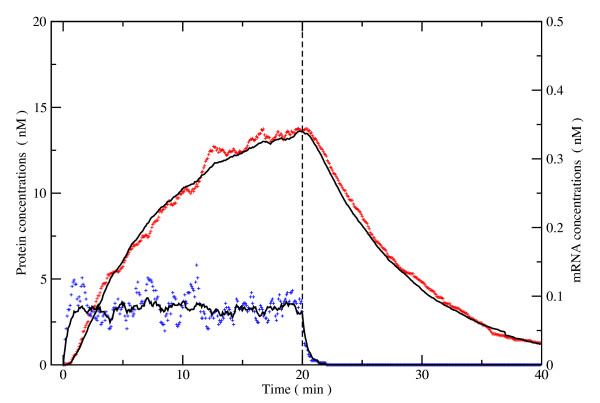
**Example timecourse**. Concentrations of protein in the nucleus (red) and mRNA in the cell (blue), the scatter plots show the data of a single simulation, black lines are averages over 10 runs.

Figure 5 presents the average concentrations of protein in the nucleus and mRNA molecules in the cell over the time course of the simulation. Data obtained using the Smoluchowski approach as modified by Andrews and Bray are virtually indistinguishable from the ones produced using the present approach and are not shown. As expected, a delay occurs before the protein concentration in the nucleus increases. The mRNA concentration quickly reaches a maximum value of ~0.06 nM over the first 2 minutes. The nuclear protein concentration increases sharply over the first 10 minutes and then starts to plateau at values of ~13 nM. At *t *= 20 min the gene is turned off and the mRNA concentration quickly falls off (half-life ~1 min) with the protein concentration quickly following (half-life ~5 min) due to degradation. Figure 5 also shows the timecourses of an individual run. As expected, individual runs present stochastic behaviour characteristic of such biological systems. These dynamics are typical of what one would expect of such a system [31].

## Discussion & conclusion

We have presented a formal, theoretically sound framework that provides reliable and accurate simulations of the diffusion-reaction process for biological systems. We compared it with the methods of Smoluchowski [22] and its extension by Andrews and Bray [17]. Figure 1 illustrates the different approaches compared in this study. The first case (A) is the original Smoluchowski approach. In this approach, at each short timestep *δt *in the diffusion process the distance between the chemical entities is checked and if they come into close proximity (distance *d *<*σ*_*b*_) the two entities are said to have reacted together. The downside of the approach is that many diffusion steps need to be computed to simulate the reaction kinetics accurately. The second approach (B) is that of Andrews and Bray [17]. In their scheme, the reaction radius, *σ*_*b*_, is adjusted so that the correct reaction kinetics are reproduced for timesteps Δ*t *≥ 100 × *δt*. This approach produces an efficient algorithm that yields the correct reaction kinetics while using larger timesteps. Finally, (C) illustrates the present approach where the reaction radius is replaced by a smooth interaction probability. The two entities are considered to diffuse freely during the timestep Δ*t *thereby producing a probability *P*^*AB *^(*d*, Δ*t*) of interaction.

Although differences were expected to appear between the Andrews and Bray and the current approach in certain circumstances (such as low reactant concentrations, or in the presence of concentration gradients), the results indicate that the reactions rates produced by both methods converge. This is thought to be essentially due to the averaging that takes place as the number of interactions increases. Hence the two methods are for practical purposes equivalent (*p *> 0.55). It cannot be ruled out, however, that differences will appear for more complex systems. For example, in the context of reversible reactions, recombination effects might be best modelled using a probability based method. Overall, the Andrews and Bray method for simulating diffusion-reaction processes appears robust at low concentration and gradient effects. However, a possible improvement on this method would be the analytical derivation of the radius of reaction for long timesteps, in place of its present approximation. The Andrews and Bray method was consistently computationally more efficient, running up to ~15% faster depending upon the system being simulated.

An in depth theoretical analysis of the diffusion-reaction approach in the context of event driven simulations has recently been published by Zon and Wolde [27]. Here again the aim is to increase the reach of present simulations by using longer timesteps. Using event driven simulations, the timestep can be increased substantially when reactive species are far apart or present at very low concentrations. However, as in the present work a limit on the length of the timestep is set by the requirement that they have to be short enough to ensure that an object can only interact with one other object during a timestep; this sets an upper limit to how large a timestep can be, and it remains to be shown whether they offer any clear computational advantage.

We have shown that the modified Smoluchowski method provides results that are indistinguishable from those produced using the much more elaborate and realistic model presented here, at a lower computational cost. The Andrews and Bray, radius-based, method thus appears to be the most simple, robust and efficient method for simulating diffusion-reaction processes currently available.

## Competing interests

The authors declare that they have no competing interests.

## Authors' contributions

ALT designed the new methodology and mathematics, PWF helped with implementation and in checking the derivations, PAB provided the initial impetus and supported the project through its different stages. All authors read and approved the final manuscript.
